# Genomic diversity and antimicrobial resistance of Vibrio cholerae isolates from Africa: a PulseNet Africa initiative using nanopore sequencing to enhance genomic surveillance

**DOI:** 10.1099/mgen.0.001586

**Published:** 2025-12-10

**Authors:** Ebenezer Foster-Nyarko, Shola Able-Thomas, Nana Eghele Adade, Rexford Adade, Jean Claude Blessa Anne, Loretta Antwi, Yaya Bah, Gifty Boateng, Heather Carleton, David Chaima, Roma Chilengi, Kalpy Julien Coulibaly, Firehiwot Abera Derra, Dwayne Didon, Cheelo Dimuna, Mireille Dosso, Momodou M. Drammeh, Sana Ferjani, Kathryn E. Holt, Rohey Jatta, John Bosco Kalule, Abdoulie Kanteh, Hortense Faye Kette, Dam Khan, N’da Kouame Nazaire Kouadio, Christine Lee, Hamakwa Mantina, Gillan Mulenga, John Mwaba, Fatou Nyang, Godfred Owusu-Okyere, Jessica Rowland, Aissatou Seck, Abdul Karim Sesay, Anthony Smith, Peyton Smith, Djifahamaï Soma, Nomsa Tau, Pierrette Landrie Simo Tchuinte, Peggy-Estelle Maguiagueu Tientcheu, Chalwe Sokoni, Sabine N'dri Vakou, Delfino Vubil

**Affiliations:** 1 Department of Infection Biology, London School of Hygiene and Tropical Medicine, London, UK; 2 Medical Research Council Unit, The Gambia at The London School of Hygiene and Tropical Medicine, Fajara, Gambia; 3 Department of Biochemistry, Cell and Molecular Biology, University of Ghana, Accra, Ghana; 4 National Public Health and Reference Laboratory, Accra, Ghana; 5 Institut Pasteur of Côte d’Ivoire, Abidjan, Côte d'Ivoire; 6 Centers for Disease Control and Prevention, Atlanta, Georgia, USA; 7 Department of Pathology, Kamuzu University of Health Sciences, School of Medicine and Oral Health, Lusaka, Zambia; 8 Zambia National Public Health Institute, Lusaka, Zambia; 9 Department of Food Science and Nutrition Research Directorate, EPHI, Addis Ababa, Ethiopia; 10 Seychelles Public Health Laboratory, Public Health Authority, Ministry of Health, Victoria, Seychelles; 11 Zambia National Public Health Institute, Lusaka, Zambia; 12 Laboratory of Microbiology, Charles Nicolle Hospital, Tunis, Tunisia; 13 Faculty of Medicine of Tunis, University of Tunis El Manar, LR99ES09, Tunis, Tunisia; 14 National Public Health Laboratories, Kotu, Gambia; 15 Department of Biotechnical and Diagnostic Sciences, Makerere University, College of Veterinary Medicine, Animal Resources and Biosecurity, Kampala, Uganda; 16 Department of Pathology and Microbiology, University Teaching Hospital, Lusaka, Zambia; 17 Association of Public Health Laboratories, Bethesda, Maryland, USA; 18 Centre for Enteric Diseases, National Institute for Communicable Diseases, Division of the National Health Laboratory Service, Johannesburg, South Africa; 19 Department of Medical Microbiology, School of Medicine, Faculty of Health Sciences, University of Pretoria, Pretoria, South Africa; 20 Departement de Biochimie Microbiologie, Université Joseph KI-ZERBO, Ouagadougou, Burkina Faso; 21 Faculty of Health Sciences, University of the Witwatersrand, Johannesburg, South Africa; 22 Department of Hygiene and Environment, Microbiology Section, Centre Pasteur of Cameroon, Yaounde, Cameroon; 23 Centro de Investigação em Saúde de Manhiça (CISM), Manhica, Mozambique

**Keywords:** cholera, foodborne infections, PulseNet Africa, Oxford Nanopore Technology Sequencing

## Abstract

**Objectives.** *Vibrio cholerae* remains a significant public health threat in Africa, with antimicrobial resistance (AMR) complicating treatment. This study leverages whole-genome sequencing (WGS) of *V. cholerae* isolates from Côte d’Ivoire, Ghana, Zambia and South Africa to assess genomic diversity, AMR profiles and virulence, demonstrating the utility of WGS for enhanced surveillance within the PulseNet Africa network.

**Methods.** We analysed *Vibrio* isolates from clinical and environmental sources (2010–2024) using Oxford Nanopore sequencing and hybracter assembly. Phylogenetic analysis, MLST, virulence and AMR gene detection were performed using Terra, Pathogenwatch and Cloud Infrastructure for Microbial Bioinformatics platforms, with comparisons against 118 global reference genomes for broader genomic context.

**Results.** Of 79 high-quality assemblies, 67 were confirmed as *V. cholerae*, with serogroup O1 accounting for the majority (43 out of 67, 67%). ST69 accounted for 60% (40 out of 67) of isolates, with 8 sequence types identified overall. Thirty-seven isolates formed distinct sub-clades within AFR12 and AFR15 O1 lineages, suggesting local clonal expansions. AMR gene analysis revealed genes associated with resistance to trimethoprim in 96% of isolates and genes associated with resistance to quinolones in 83%, while genes associated with resistance to azithromycin, rifampicin and tetracycline remained low (≤7%). A significant proportion of the serogroup O1 isolates (41 out of 43, 95%) harboured resistance genes in at least 3 antibiotic classes.

**Conclusions.** This study highlights significant genetic diversity and AMR prevalence in African *V. cholerae* isolates, with expanding AFR12 and AFR15 clades in the region. The widespread presence of genes associated with resistance to trimethoprim and quinolones raises concerns for treatment efficacy, although azithromycin and tetracycline remain viable options. WGS enables precise identification of species and genotyping, reinforcing PulseNet Africa’s pivotal role in advancing genomic surveillance and enabling timely public health responses to cholera outbreaks.

**Impact Statement**

Cholera remains a significant public health challenge in Africa, disproportionately affecting the region due to the ongoing transmission of *Vibrio cholerae* O1 and the emergence of antimicrobial resistance (AMR). This study demonstrates the utility of Oxford Nanopore Technology sequencing in providing high-resolution insights into the genomic diversity, transmission dynamics and AMR profiles of *V. cholerae* isolates across Africa. By generating and analysing whole-genome sequences, we identified clonal expansions of established lineages and dissemination of AMR genes. These findings contribute to a deeper understanding of the epidemiology and evolution of *V. cholerae* in Africa, informing targeted intervention strategies.

Furthermore, the study highlights the growing threat posed by AMR among *V. cholerae* isolates, including persisting genes associated with resistance to key therapeutic antibiotics, such as quinolones and trimethoprim, which could undermine current treatment protocols. Despite this, the absence of genes associated with resistance to azithromycin and rifampicin among the O1 isolates suggests that these drugs may remain viable treatment options, offering a critical avenue for preserving treatment efficacy.

This research also underscores the importance of sustained genomic surveillance, capacity building and regional collaboration to mitigate the public health impact of cholera and other foodborne pathogens. By leveraging whole-genome sequencing technologies and training initiatives, such as the PulseNet Africa genomics workshop, this study provides a framework for strengthening regional capacities to detect, monitor and respond to cholera outbreaks and the spread of AMR. These efforts align with the African Union and Africa CDC’s strategic priorities on health security and AMR, contributing to improved public health systems and cholera control across the continent.

## Data Summary

All supporting data and protocols have been provided within the article or as supplementary data files. The Oxford Nanopore Technology reads have been deposited under BioProject accession PRJNA1192988, while the high-quality *Vibrio* spp. assemblies have been shared via figshare [Foster-Nyarko, Ebenezer (2024). Genomic Diversity and Antimicrobial Resistance of Vibrio spp. Isolates from Africa: A PulseNet Africa Initiative Using Nanopore Sequencing to Enhance Genomic Surveillance. figshare. Dataset. https://doi.org/10.6084/m9.figshare.27941376.v1]. Individual accession numbers for these reads and Biosample IDs are provided in File S2, available in the online Supplementary Material. The accession numbers for the 118 reference genome assemblies included in our analysis are also provided in File S3.

## Introduction

Cholera, caused by toxigenic strains of *Vibrio cholerae* O1 and O139, presents with mild to potentially fatal acute watery diarrhoea [1,3]. The current seventh cholera pandemic, primarily due to the O1 biotype El Tor, disproportionately affects Africa, which bears the majority of the global disease burden [2]. Despite its longstanding presence since resurging in 1970, the transmission mechanisms of *V. cholerae* in Africa remain poorly understood. This knowledge gap is exacerbated by evolving antimicrobial resistance (AMR) patterns, climate change and increased global mobility [3,7].

Recent genomic analyses have revealed multiple introductions of the *V. cholerae* serogroup O1 biotype El Tor strain from Asia to Africa, with at least 13 distinct sublineages identified since 1970, indicating a continued pattern of introduction from Asia [8,11]. Understanding the genomic epidemiology and AMR profiles of *V. cholerae* in Africa is essential for developing effective surveillance and intervention strategies. Conventional methods like culture and antimicrobial susceptibility testing, PCR and point-of-care testing have been instrumental in providing real-time information on circulating serotypes and AMR patterns in endemic countries in sub-Saharan Africa. However, these methods offer limited resolution for tracking transmission pathways and understanding the evolutionary dynamics of circulating *V. cholerae* clones. Whole-genome sequencing (WGS) technologies, including Illumina and Oxford Nanopore Technology (ONT), provide enhanced resolution for characterising outbreak isolates, identifying genetic determinants of AMR, tracing transmission networks and elucidating the persistence and evolution of *V. cholerae* in endemic regions. Such genomic insights are vital for informing more effective cholera control strategies and public health responses.

**Fig. 1.**
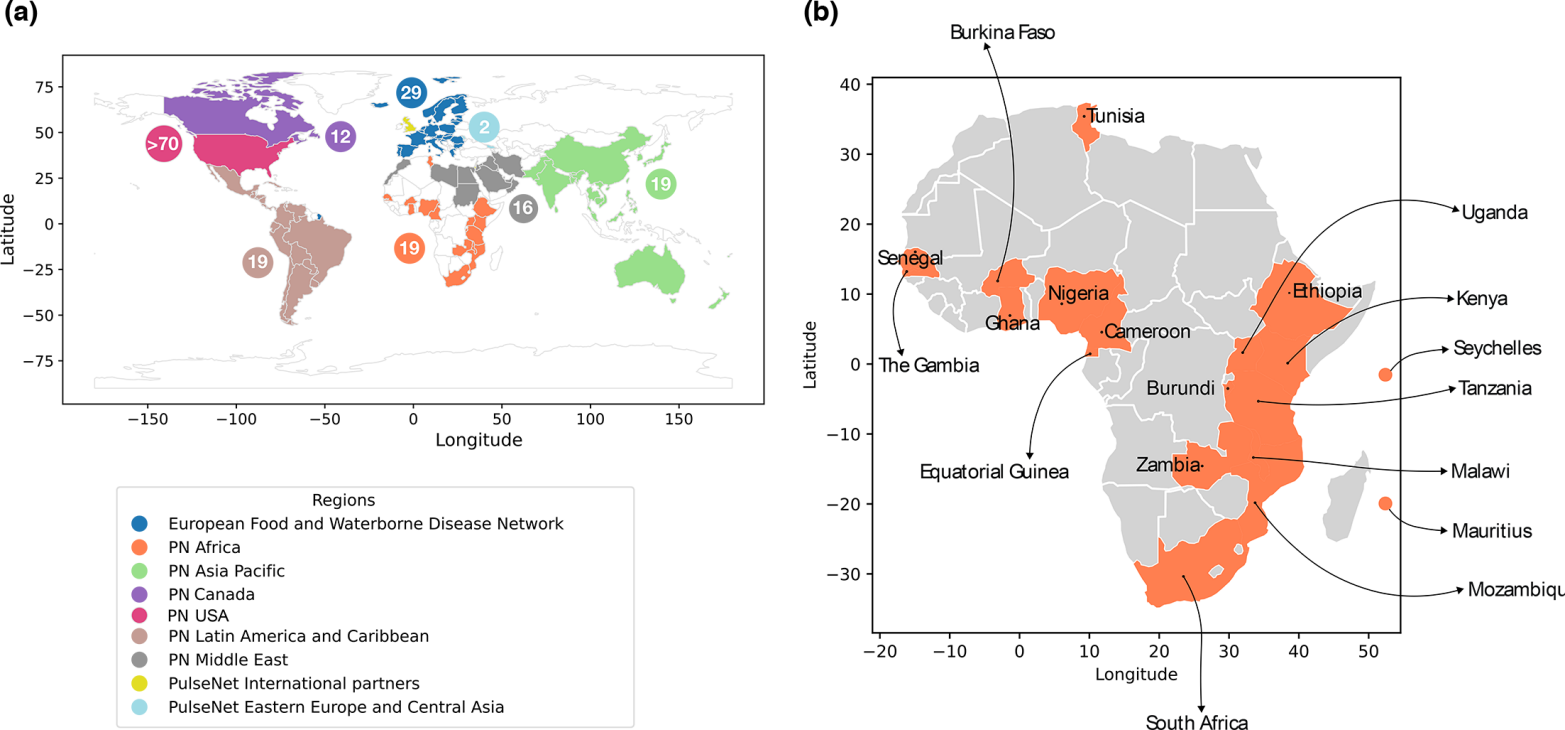
PulseNet International Regions and PulseNet Africa Member Countries. (**a**) A depiction of PulseNet International Regions, colour-coded as follows: blue, European Food and Waterborne Disease Network; orange: PulseNet Africa; green: PulseNet Asia Pacific; purple: PulseNet Canada; brown: PulseNet Latin America and Caribbean; grey: PN Middle East; light yellow: PulseNet International Partners; light cyan: PulseNet Eastern Europe and Central Asia. The participating countries representing Africa, Asia Pacific, Eastern Europe and Central Asia, the European Food and Waterborne Disease Network, Latin America and Caribbean, Middle East and PulseNet International Partners are denoted in the corresponding colour-coded bubbles. (**b**) This panel zooms in on the PN Africa region and highlights its member countries.

Given its global distribution, international transmission and endemicity across most of sub-Saharan Africa, *V. cholerae* has been a priority pathogen for PulseNet Africa since the network’s inception in 2010. PulseNet Africa is a regional collaboration of public health laboratories dedicated to tracking food- and water-borne disease outbreaks, including AMR. Alongside *V. cholerae*, PulseNet Africa monitors *Salmonella enterica*, diarrhoeagenic *Escherichia coli*, *Shigella* spp., *Campylobacter* spp. and *Listeria monocytogenes* across 19 African countries (Fig. 1). It operates within the global PulseNet International network, collaboratively connecting over 108 countries in 8 regions to monitor and control foodborne disease outbreaks.

The World Health Organization (WHO) recognises the critical role of WGS in enhancing global public health strategies, particularly for foodborne pathogens and AMR surveillance [12,14]. In the context of foodborne pathogen surveillance, the WHO highlights the utility of WGS in outbreak investigations, response efforts and routine monitoring. WGS facilitates precise pathogen identification, source tracing and an improved understanding of genetic diversity. This precision is especially significant within a One Health framework, where human, animal and environmental health are interconnected. By enabling more effective tracking of foodborne diseases, WGS strengthens public health interventions and supports global health security [12,13].

Additionally, WGS offers invaluable insights into resistance mechanisms, as well as the emergence and spread of AMR, informing evidence-based policy development for AMR control [13]. As countries advance toward cholera elimination, WGS analyses can be particularly impactful, distinguishing between rare, newly detected cases and those arising from previously circulating endemic strains. Thus, the integration of WGS into national and international surveillance systems is essential for enhancing precision, enabling swift outbreak responses and promoting accurate cross-border data sharing.

Recently, PulseNet International adopted WGS in place of PFGE and multiple-locus variable number tandem repeat analysis (MLVA) for molecular sub-typing, owing to the superior resolution of WGS, which has substantially enhanced genomic epidemiological surveillance and tracking of AMR genes across bacterial populations [15,18]. As molecular capacity continues to expand across the African continent, the implementation of standardised genomic surveillance for high-priority pathogens has become increasingly feasible [19]. Additionally, the upcoming discontinuation of support for BioNumerics – the primary software for analysing data generated through PFGE, MLVA and WGS – beyond 2024 presents a timely opportunity to evaluate and adopt alternative analysis tools that can address the diverse needs of the network.

To strengthen the genomic sequencing capabilities of participating laboratories in PulseNet Africa, we organised a hands-on genomics workshop focusing on ONT sequencing in collaboration with the Medical Research Council Unit The Gambia at London School of Hygiene and Tropical Medicine (MRCG at LSHTM), Theiagen Genomics LLC, the Association for Public Health Laboratories (USA) and the US Centers for Disease Control and Prevention (CDC). This workshop, held in July 2024 at the MRCG at LSHTM, provided both wet lab training on ONT sequencing and dry lab training for genomic data analysis using the Terra platform. Participants brought DNA from archived, unsequenced isolates identified as *V. cholerae* from outbreaks or routine surveillance in their countries. These samples were used to train participants on PulseNet International’s protocols for ONT sequencing of food- and water-borne pathogens. The workshop culminated in collaborative data analysis, where participants interpreted the genomic data to gain insights into the transmission dynamics and AMR profiles of *V. cholerae* across Africa. We hypothesisfged that *V. cholerae* isolates from various African regions would exhibit distinct transmission events, lineages and AMR profiles.

Our efforts align with the African Union and Africa CDC’s strategic priorities on health security and AMR. By elucidating the genomic diversity, evolution and AMR dissemination of *V. cholerae* in Africa, this study provides crucial insights into the regional and global emergence of multi-drug-resistant strains. The genomics workshop and this study represent significant steps toward enhancing the capacity for genomic sequencing of *V. cholerae* and other critical pathogens within Africa. Additionally, establishing a skilled professional network will help foster ongoing collaboration and support, potentially contributing to long-term improvements in public health systems across the continent.

## Methods

### Archived *V. cholerae* isolates analysed in this study

**Fig. 2.**
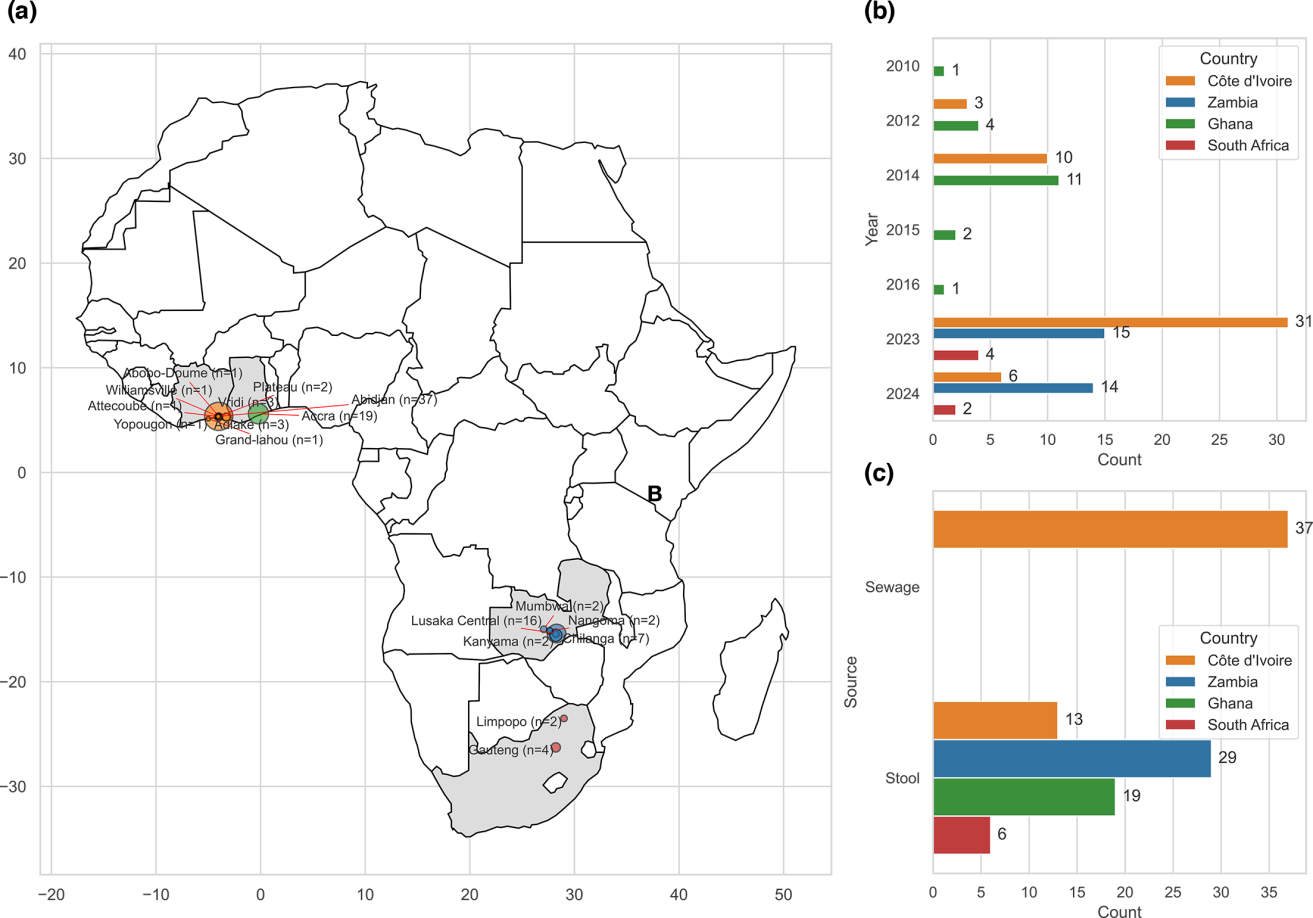
Geographical distribution, collection year and sample source of the isolates analysed in this study. (**a**) Geographical distribution of the isolates analysed in this study. The map highlights the locations where the samples were collected. (**b**) The number of isolates collected per year is displayed, showing the temporal distribution of sample collection across different years per sampling site, as depicted in the legend. (**c**) The sample sources are categorised and plotted to represent the proportion of samples collected from outbreak (stool) or non-outbreak (sewage) situations.

We generated WGS data from 104 archived isolates initially identified as *V. cholerae* by conventional microbiological methods (File S1), available in the online Supplementary Material) from 4 countries: Côte d'Ivoire (*n*=50), Ghana (*n*=19), Zambia (*n*=29) and South Africa (*n*=6), between 2010 and 2024 (Fig. 2a) – from stool (*n*=67) or sewage (*n*=37) samples. These isolates represented archived isolates derived from outbreaks or environmental surveillance (File S1). Metadata associated with the isolates included the year of collection and geographical information (i.e. patient residential province or city) (File S2).

### Global context isolates

To determine where our isolates sit within the global phylogeny, we included publicly available sequence data from 118 global reference strains of *V. cholerae* (File S3), representing the known diversity of *V. cholerae* serogroup O1 [8,11,20], collected between 1970 and 2023.

### DNA extractions and ONT sequencing

DNA extractions were performed in PulseNet-participating laboratories using standard protocols specific to each laboratory. In Zambia, extractions utilised the Invitek DNA extraction kit (Invitek Diagnostics, Germany), while isolates from Ghana were extracted using the Qiagen DNeasy blood and tissue kit (Qiagen Johannesburg). Nucleic acid extractions in Côte d’Ivoire were performed with the Spin-X viral DNA/RNA extraction kit (SD Biosensor, Republic of Korea), and in South Africa, the QIAmp DNA mini kit (Qiagen Johannesburg) was used. Library preparation followed PulseNet International’s standard operating procedures using ONT’s rapid barcoding kit.

All isolates, except those from South Africa, were sequenced during the training workshop at MRCG at LSHTM. The libraries were loaded onto 11 MinION R10 flowcells and sequenced on the GridION, Mk1cs or Mk1bs platforms. In South Africa, sequencing was conducted at the National Institute for Communicable Diseases (NICD), and the ONT reads were basecalled using the same protocol employed at MRCG at LSHTM. The sequences from all locations were subsequently combined for analysis.

The basecalling utilised ONT’s basecaller, Dorado v0.7.2, with the SUPer-accuracy model (SUP) v4.3.0, via the high-performance computing clusters at MRCG at LSHTM or NICD. Subsequent bioinformatic analyses were conducted on the Terra platform (https://terra.bio/), the Cloud Infrastructure for Microbial Bioinformatics (CLIMB) [23] and Pathogenwatch [24].

### Bioinformatics analysis

The analysis on Terra utilised the TheiaProk_ONT_PHB v.2.1.0 and TheiaProk_FASTA_PHB v2.2.0 workflows. Initially, reads were quality-checked with FastQC [25]. Assemblies were then generated on CLIMB using Hybracter v0.7.3 [26] via CLIMB [23]. The hybracter assemblies were then uploaded to Terra, where TheiaProk_FASTA_PHB v2.2.0 was deployed to characterise the genome assemblies. This included speciation with Gambit v1.0.0 (database v2.0.0-20240628.gdb) [27], identification of *omp*W and t*ox*R loci and *Vibrio* characterisation using Abricate v1.0.1 [28], MLST using the mlst software v2.23.0 (https://github.com/tseemann/mlst) and plasmid identification with PlasmidFinder v2.1.6 [29].

Next, we uploaded the assemblies to Pathogenwatch (https://pathogen.watch/), where a collection was created and neighbour-joining trees were reconstructed using a concatenated alignment of 27,336 genes which constitute the core gene library for *V. cholerae* in Pathogenwatch. PopPUNK [30] was deployed to determine the placement of our study isolates as belonging to the current pandemic lineage using the PopPUNK clusters implemented within Vibriowatch [24]. PopPUNK classifies user-uploaded sequences to 1 of 41 stable clusters corresponding to the previously named *V. cholerae* lineages. For example, cluster 1 denotes isolates belonging to the current pandemic lineage (7PET), clusters 2 and 7 are part of Lineage 3b and clusters 75 and 76 encompass strains that are part of ELA-1 [24]. If a novel lineage is encountered, PopPUNK assigns cluster VC1870. For ONT assemblies, it is imperative to ascertain that the isolate truly belongs to a novel lineage and that sequencing errors are not hampering the PopPUNK analysis. Consequently, for each study isolate typed as VC1780 by PopPUNK or not belonging to previously characterised lineages, we created a Vibriowatch collection containing the study isolate and some contextual isolates from the Chun *et al*. [31] collection, available in Pathogenwatch [24]. This collection encompasses isolates from the 7PET lineage and the other known *V. cholerae* lineages [31]. The neighbour-joining trees generated by Vibriowatch were then inspected to determine the clades the isolate fell into. Additionally, Pathogenwatch calls AMR genes using an in-house AMR search tool by scanning against a curated AMR gene library hosted on Pathogenwatch.

To contextualise the AMR genes and mutations found among the *V. cholerae* isolates against what pertains to the global *V. cholerae* O1 population, we searched for all *V. cholerae* O1 isolates in Pathogenwatch and downloaded the associated AMR genotype predictions and analysed the resulting data frame using Python’s pandas library v2.0.3. via Jupyter Notebook.

We then combined the *V. cholerae* ST69 sequences generated in this study with 118 publicly available representative sequences from the known 7PET *V. cholerae* sequences and performed a whole-genome alignment of assemblies using the alignment-free mode implemented in the Split K-mer Analysis (SKA2) v0.3.7 [32] – a rapid and accurate approach which utilises k-mers to detect variation between samples. SNPs detected by SKA have been shown to be comparable to those determined by read alignment-based methods such as Snippy [32,34]. A maximum-likelihood phylogeny was inferred from the resulting SNP alignment using RAxML v8.2.12 [35] with the generalised time-reversible substitution model and 1,000 bootstrap replicates. The phylogenetic tree was visualised using FigTree v1.4.4 and annotated in R v4.1.0 (2021-05-18) with the ggtree package v3.0.4 and Adobe Illustrator v28.0.

Among the study isolates from Ghana, a single isolate, ‘490–12’, had been previously sequenced using Illumina (accession: ERR1953608; Opintan *et al.* [36]). We utilised this publicly available data to benchmark the quality of our corresponding ONT assembly by assembling with Shovill v1.1.0 [37] and calculating the pairwise SNP distance between the ONT assembly generated in this study and that from the Illumina reads using SKA2 v0.3.7 [32].

## Results

In total, we sequenced 67 clinical and 37 environmental cultured isolates previously identified as *V. cholerae*. The environmental isolates were collected exclusively from sewage samples in Abidjan, Côte d’Ivoire, between 2023 and 2024. For the clinical isolates, samples from Côte d’Ivoire were spread over seven towns or cities; those from Zambia originated from five sub-counties, while those from Ghana were sourced from the nation’s capital, Accra. The isolates from South Africa were derived from Limpopo and Gauteng (Fig. 2b, c and File S2).

Since ONT sequencing for *Vibrio* is new to PulseNet International, and QC thresholds for ONT data are not yet established, we aimed to assess the assembler’s performance at varying read depths. We initially set a minimum read threshold of 5,000 reads, independent of sequencing depth, hypothesising that fewer reads would be sufficient for high coverage due to the longer ONT read lengths. All samples met this threshold and were assembled using Hybracter [26]. The resulting assemblies were then examined with Bandage, which showed that samples with read coverage below 20× generally produced highly fragmented assemblies (>10 contigs) and low assembly coverage (<20× mean depth), except for three samples (CO42-15, CIV34 and ZA29) with read coverages of 8×, 13× and 18×, respectively, which yielded fully circularised chromosomes. Therefore, in the downstream analysis, we only included samples with fully assembled chromosomes or a mean read depth and assembly coverage of ≥20×, excluding those below this threshold. A total of 25 isolates, comprising 19 from stool and 6 from sewage, fell below this threshold and were excluded, leaving a final dataset of 79 isolates. Detailed read and assembly metrics are presented in Fig. S1 and File S2.

Of the 25 isolates excluded from the final dataset due to insufficient read depth or assembly quality, 19 were from stool samples, and 6 were from sewage. Despite not meeting the QC thresholds, some isolates could still be speciated: 17 were identified as *V. cholerae*, 1 as *Aeromonas caviae* and 1 as *Pseudomonas aeruginosa*, while 3 samples remained unidentified, and 3 failed to assemble altogether. Geographically, these excluded samples were distributed between Zambia [11], Côte d’Ivoire [11] and South Africa [3].

Of the 79 high-quality assemblies that constitute the final dataset, speciation with Gambit [27] and Pathogenwatch’s Speciator [38] were in agreement and revealed 67 out of 79 (85%) isolates as *V. cholerae*, 2 out of 79 (3%) each as *V. fluvialis* and *V. navarrensis* and 1 out of 79 (1%) as *V. furnissii*. The *V. furnissii* isolate was recovered from a clinical (stool) specimen in Vridi, Côte d’Ivoire, while the *V. fluvialis* and *V. navarrensis* isolates were all sourced from sewage samples in Abidjan (File S2). All isolates confirmed by WGS as non-cholerae *Vibrio* spp. resulted in complete circularised assemblies.

The remainder of the isolates typed as non-*Vibrio* species was as follows: *Enterobacter hormaechei* (4 out of 79, 5%), *Aeromonas enteropelogenes* (2 out of 79, 3%) and *Klebsiella quasipneumoniae* (1 out of 79, 1%).

The 67 isolates confirmed by WGS as *V. cholerae* comprised *n*=46 from clinical (outbreak) cases (stool) and *n*=21 sewage isolates. Of these, 46 had two completely circularised chromosomes each, comprising *n*=30 clinical isolates and *n*=16 from sewage. The assembly metrics are depicted in Fig. S1.

The individual sample accessions for the high-quality *V. cholerae* isolates are provided in File S2, and the *Vibrio* spp. assemblies are shared via figshare (https://doi.org/10.6084/m9.figshare.27941376.v1). The rest of the results focus on the *V. cholerae* isolates.

### Phylogenetic diversity of the study strains

We recovered 8 sequence types overall, with ST69 accounting for the majority (40 out of 67, 60%), followed by non-typeable (12 out of 67, 18%), ST85 and ST75-2LV (2 out of 67, 3% each) and ST289, ST832, ST357, ST366 and ST69-4LV (1 out of 67, 2% each). Six isolates were assigned one or more novel alleles out of the seven housekeeping genes utilised in the MLST, indicating potentially novel STs.

Most (43 out of 46, 93%) of the clinical isolate genomes were identified as *V. cholerae* serogroup O1 and biotype El Tor, consistent with the serological results. The majority of *V. cholerae* O1 isolates belonged to ST69 and were assigned to the VC1 cluster by PopPUNK, indicating their association with the current pandemic lineage. Notably, two exceptions were observed: the ST75-2LV isolates from South Africa, which clustered within the pre-7PET/‘Gulf-Coast-like’ clade [39,41] (Fig. S2). This clade is thought to represent close relatives of the most recent common ancestor of the 7PET clade [24]. The nearest relative to the two ST75-2LV strains was isolate 2740-80, a US Gulf Coast *V. cholerae* O1 clone previously associated with the 2018–2020 cholera outbreak in South Africa [42]. In addition, two non-O1 clinical isolates (‘CIV50’ and ‘CIV55’), collected from Abobo Doumé and Williamsville, Côte d’Ivoire, respectively, were identified as belonging to ST85 and were assigned to the VC995 cluster. A single environmental isolate from ST366 was assigned to VC506. The remaining non-O1 environmental isolates were grouped into VC1870 by PopPUNK, further underscoring their genomic diversity.

Our phylogenetic analysis with representative global reference strains places the study isolates within the context of the global *V. cholerae* O1 population, revealing the circulation and local evolution of established 7PET sublineages in West and Southern Africa. The majority of clinical isolates (40 out of 46, 87%) clustered within three known sublineages – AFR9, AFR12 and AFR15 – forming distinct, geographically linked clades indicative of ongoing clonal expansions.

**Fig. 3.**
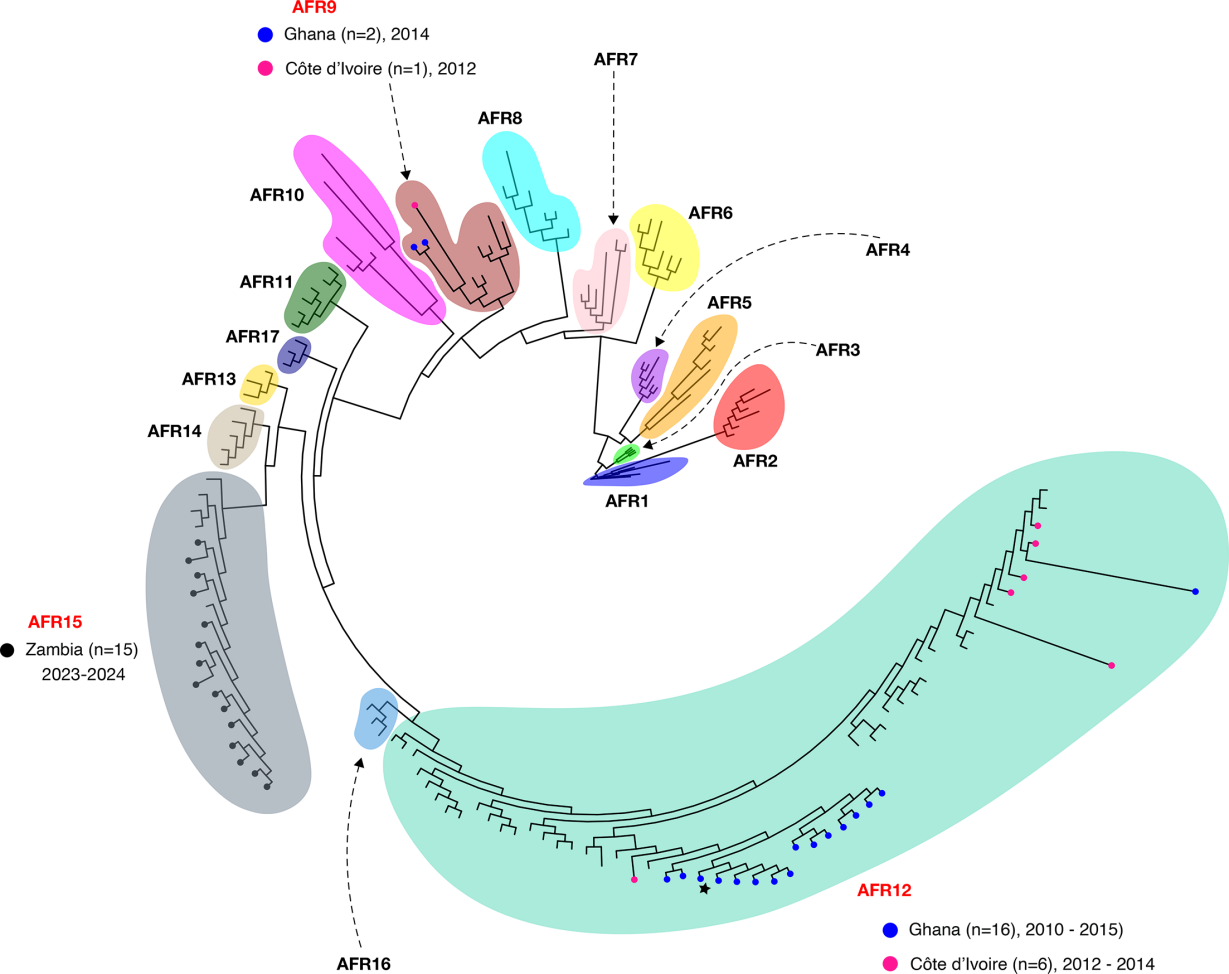
A maximum-likelihood tree depicting the population structure of the *V. cholerae* O1 study isolates (*n*=40), along with 118 publicly available genomes representing the known diversity of *V. cholerae* O1. The phylogenetic tree was reconstructed using RAxML v8.2.12 [35] with 1,000 bootstrap replicates, based on a reference-free whole-genome alignment generated with SKA2 [32,34]. The branch lengths indicate genetic distances between samples. The clades are highlighted and annotated to show the previously known lineages (e.g., AFR1 and AFR2), with the study samples distinguished from the representative reference genomes by the black circular tips (Zambian isolates), deep pink (isolates from Côte d’Ivoire) or blue (Ghanaian isolates). Isolate ‘490-12’, which had been previously sequenced using Illumina (accession: ERR1953608; Opintan *et al*., 2021 [36]), is denoted by a star. The reference genomes are tip-less. Each colour denotes a unique *V. cholerae* O1 lineage. Where they occur, the distribution of study isolates found in each lineage is displayed next to the lineage name. Distinct clonal expansions with AFR9, AFR12 and AFR15 arising from this study are denoted in red.

For instance, *n*=22 isolates from Ghana and Côte d’Ivoire (2010–2015) formed a large clade within the AFR12 sublineage, which was previously identified in Cameroon and Nigeria and thought to have been directly imported from South Asia [11]. Similarly, isolates from Zambia (2023–2024) formed specific clusters within the AFR15 sublineage that recently caused a large outbreak in South Africa [8]. Likewise, isolates ‘17-14’ and ‘C413-14’, collected in Accra in 2014, along with isolate CIV57 derived from Adiaké, Côte d’Ivoire, in 2012, clustered with reference genomes belonging to T9/AFR9, indicating genetic similarity to these known *V. cholerae* O1 strains (Fig. 3).

While our analysis demonstrates clear evidence of local transmission and clonal expansion within African sublineages, the absence of recent Asian sequences in our phylogenetic analysis limits our ability to definitively rule out additional introductions from Asia during the study period.

The resolution of these fine-scale transmission patterns using ONT data is supported by direct benchmarking against Illumina sequencing. A comparison of our ONT data with matched, publicly available Illumina reads for isolate 490-12 (published in [36]) revealed a near-identical assembly (1-SNP difference), demonstrating the high quality and reliability of our ONT workflow for phylogenetic analysis (File S5).

### Prevalence of acquired resistance genes and resistance mutations

A limitation of our study is the absence of phenotypic antimicrobial susceptibility data. Due to logistical constraints, it was not feasible to conduct susceptibility testing; therefore, our analysis was restricted to the detection of AMR genotypes from the WGS data.

**Fig. 4.**
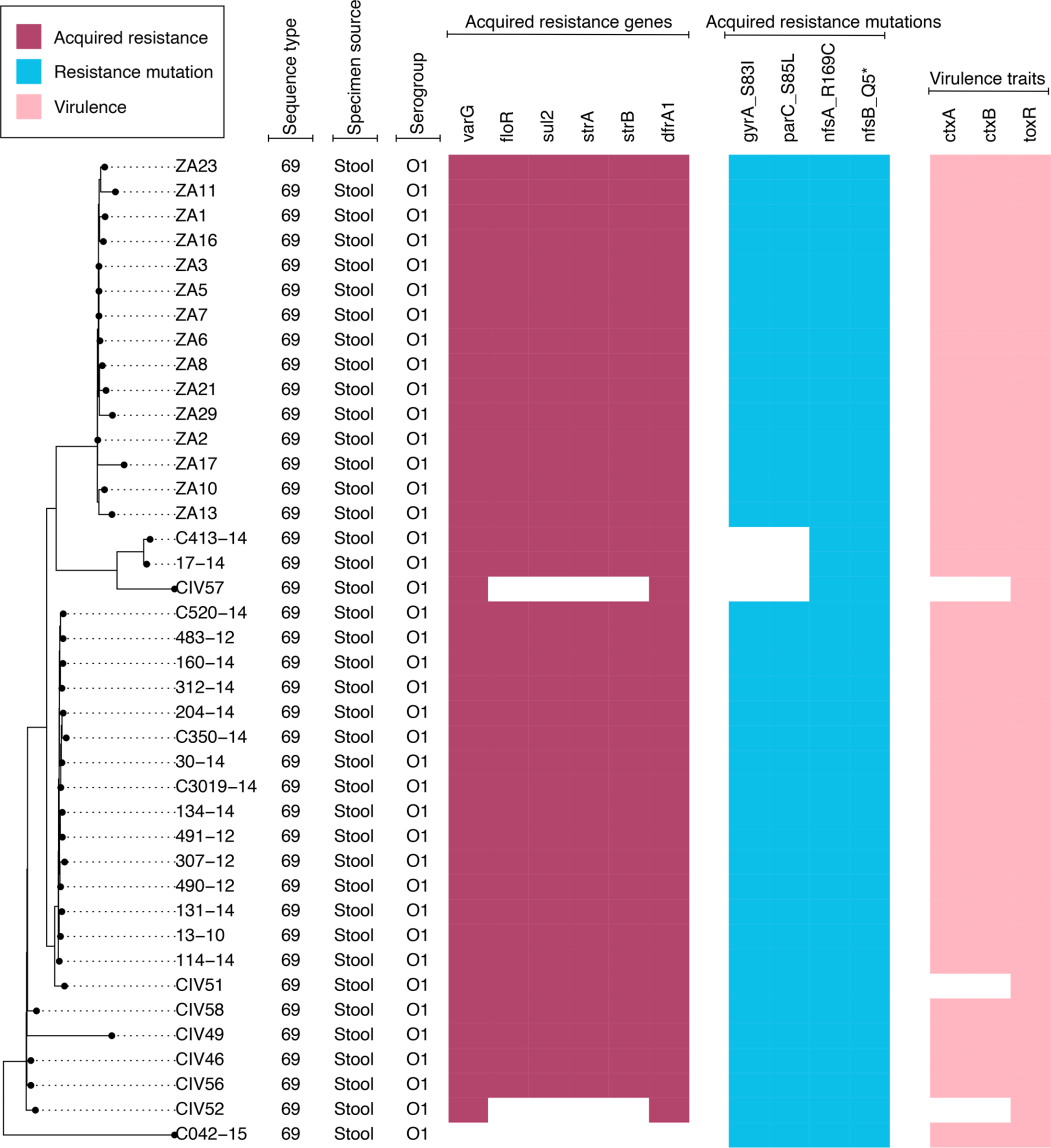
A neighbour-joining phylogenetic tree of the study *V. cholerae* O1 ST69 isolates (*n*=40) with AMR genes and virulence annotations. The phylogenetic tree depicts the evolutionary relationships among the study isolates, as determined by phylogenetic inference. The tree was reconstructed via Pathogenwatch [24], based on a concatenated alignment of 1,972 genes (2,172,367 bp) representing the core gene library for *V. cholerae* in Pathogenwatch. Each tip of the tree denotes a unique isolate, coloured by the geographic origin or source of the isolate (displayed in the legend). The heatmap visualises the presence of acquired AMR genes (raspberry), resistance mutations (blue raspberry) and virulence factors (light pink) (File S6). The resistance genes, grouped by their classes, are as follows: carbapenems (*var*G), chloramphenicol (*flo*R), aminoglycosides (*str*A, *str*B) and trimethoprim (*dfr*A1 and *dfr*A). Resistance mutations were as follows: quinolone resistance mutations (*gyr*A_S83I and *par*C_S85L) and nitrofuran (*nfs*A_R169C and *nfs*B_Q5*).

**Fig. 5.**
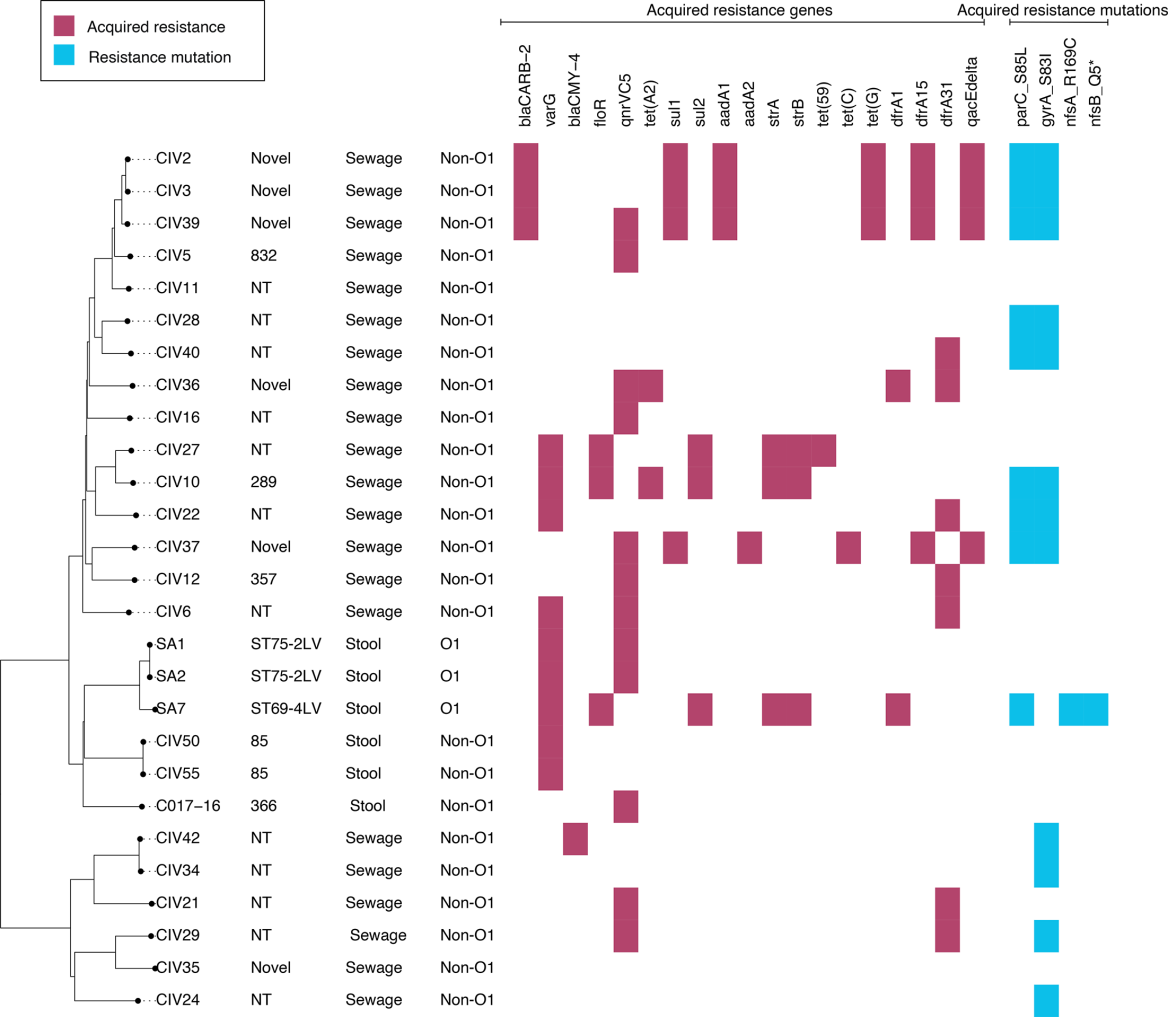
A neighbour-joining phylogenetic tree of the study *V. cholerae* non-ST69 isolates (*n*=27) with AMR genes and virulence annotations. The phylogenetic tree depicts the evolutionary relationships among the study isolates, as determined by phylogenetic inference. The tree was reconstructed via Pathogenwatch [24], based on a concatenated alignment of 2,736 genes (2,172,367 bp) representing the core gene library for *V. cholerae* in Pathogenwatch. Each tip of the tree denotes a unique isolate, coloured by the geographic origin or source of the isolate (displayed in the legend). The heatmap visualises the presence of acquired AMR genes (raspberry) and resistance mutations (blue raspberry). The resistance genes, grouped by their classes, are as follows: penicillins (*bl_a_*_CARB-2_), carbapenems (*var*G), third-generation cephalosporins (*bla*_CMY-4_), chloramphenicol (*cat*B9, *flo*R), sulphonamides (*sul*1, *Sul*2), aminoglycosides (*aad*A1, *aad*A2, *str*A, *str*B), tetracyclines [*tet*(C), *tet*(G), *tet*(59)], trimethoprim (*dfr*A1, *dfr*A15, *dfr*A31) and antiseptic (*qa*CEdelta). Resistance mutations were detected for quinolones as follows: *gyr*A_S83I and *par*C_S85.

The prevalence of genes encoding resistance to various antibiotics among the *V. cholerae* study isolates is depicted in Figs4  5. Trimethoprim resistance genes exhibited the highest prevalence, with 64 out of the 67 isolates (96%) harbouring at least 1 of 4 variants of the *dfrA* gene (Figs4  5). *dfr*A1 occurred predominantly in the clinical isolates (40 out of 46, 93%) (Fig. 4). The environmental *V. cholerae* isolates, on the other hand, mainly harboured *dfr*A31 (7 out of 24, 29%), along with *dfr*A15 (4 out of 24, 17%) and *dfr*A1 (2 out of 24, 8%) (Fig. 5). Conversely, some clinically relevant antibiotics demonstrated little or no resistance determinants among the study isolates. Notably, no determinants associated with azithromycin or rifampicin resistance were observed. Low prevalence rates were also seen for the antiseptic resistance gene *qac*Edelta, which occurred exclusively among the environmental strains (4 out of 24, 16% of isolates). Similarly, tetracycline resistance genes occurred only among the environmental isolates, echoing findings from Algeria, Central African Republic, Kenya and Malawi, indicating widespread susceptibility of *V. cholerae* O1 isolates to tetracyclines [43,46]. The genotypes included *tet*(C) in 4% of the environmental isolates (1 out of 24), *tet*(G) in 13% (3 out of 24) and *tet*(59) in 4% (1 out of 24). The ampicillin resistance gene, *bla*_CARB-2_ gene, was found in the sewage isolates only (4 out of 24, 12%).

The preponderance of the resistance determinants and mutations occurred among the *V. cholerae* O1 isolates (Fig. S3), consistent with findings from other studies [43,47]. For example, resistance determinants for chloramphenicol (*flo*R), aminoglycosides (*str*A and *str*B) and sulphamethoxazole (*sul*2) occurred in ≥88% (38 out of 43) of the O1 strains but were detected in 4–16% of the non-O1 isolates (*flo*R, 3 out of 24, 12%; *str*A, 3 out of 24, 12%; *str*B, 2 out of 24, 8%; *sul*1, 4 out of 24, 16%; respectively). Conversely, the encoding resistance to ampicillin was identified in environmental isolates only (3 out of 24, 12%), as was the *bla*_CMY-4_ gene encoding resistance to third-generation cephalosporins, which occurred in a single environmental isolate (1 out of 24, 4%) (Fig. 5).

Besides these, the *var*G resistance gene, a putative Ambler class B metallo-beta-lactamase found on the antibiotic resistance *var* regulon in *V. cholerae*, along with an antibiotic efflux pump [48], was present in 98% of the clinical *V. cholerae* O1 isolates (42 out of 43) and 29% (7 out of 24) of the environmental (non-O1) strains. However, we could not find much evidence for phenotypic carbapenem resistance due to *var*G in the literature [48,50] – a finding supported by anecdotal data from other PulseNet International partners, who confirm that this gene does not seem to confer this resistance phenotype.

In addition to resistance genes, specific mutations conferring resistance to other antibiotic classes were identified. The *gyr*A_S83I and *par*C_S85L mutations, associated with quinolone resistance, were detected in 88% (38 out of 43) of the serogroup O1 isolates. These isolates belonged to the current pandemic (7PET) clade and were distributed across the AFR12 and AFR15 lineages, consistent with the fixation of this trait in strains from these lineages [47]. By contrast, the remaining serogroup O1 isolates, which lacked these fluoroquinolone resistance mutations, comprised three ST69 strains from the AFR9 lineage and two ST75-2LV clones from the Gulf-Coast-like clade.

Mutations associated with resistance to furazolidone (*nfs*A_R169C and *nfs*B_Q5*) were detected exclusively in the ST 69 O1 isolates (40 out of 40, 100%) (Figs4  5), indicating the fixation of these mutations in the O1 ST69 population. Among non-O1 isolates, the *gyr*A_S83I and *par*C_S85L mutations were present in 50% (12 out of 24) and 33% (8 out of 24) of isolates, respectively.

Nearly all the *V. cholerae* O1 isolates (41 out of 43, 95%) harboured resistance genes in at least 3 antibiotic classes, excluding *var*G (carbapenems). Our findings mirrored a high prevalence of quinolone and nitrofuran resistance mutations and several resistance genes in the global population of *V. cholerae* O1 isolates (File S4). An analysis of *n*=4,858 *V*. *cholerae* O1 genomes hosted in Pathogenwatch [24] indicates that mutations *nfs*B*_Q5* and *nfs*A*_R169C*, conferring resistance to nitrofurans, are present in 70% of O1 isolates globally (3,419 out of 4,858 and 3,408 out of 4,858, respectively). Similarly, the *gyr*A*_S83I* and *par*C*_S85L* mutations occur in 63.96% (3,107 out of 4,858) and 56.69% (2,754 out of 4,858) of global isolates, respectively, reflecting the prevalence observed in our study, where *gyr*A and *par*C mutations were present in 88% (38 out of 43) of O1 isolates.

Our analysis revealed a near-universal distribution of *var*G in publicly available O1 strains (4,847 out of 4,858, 99.77%), supporting the hypothesis that this gene is intrinsic to the *V. cholerae* population. Other resistance genes such as *dfr*A1 and *sul*2, encoding resistance to trimethoprim, chloramphenicol and sulphamethoxazole, respectively, also exhibited similar global frequencies (*dfr*A1: 65.87% or 3,200 out of 4,858; *flo*R: 45.97% or 2,233 out of 4,858; *sul*2: 63.59% or 3,089 out of 4,858).

These findings highlight the widespread distribution of resistance traits in the global *V. cholerae* O1 population, likely driven by selective pressures and the dissemination of resistant lineages, particularly in clinical settings.

### Plasmid replicons identified and gene cargo

Among the study isolates, a single isolate was found to harbour a plasmid (identified as IncQ1-type). Importantly, this plasmid did not carry any AMR genes, suggesting that plasmids do not likely play a significant role in the dissemination of AMR in our study population.

### Prevalence of virulence traits

**Fig. 6.**
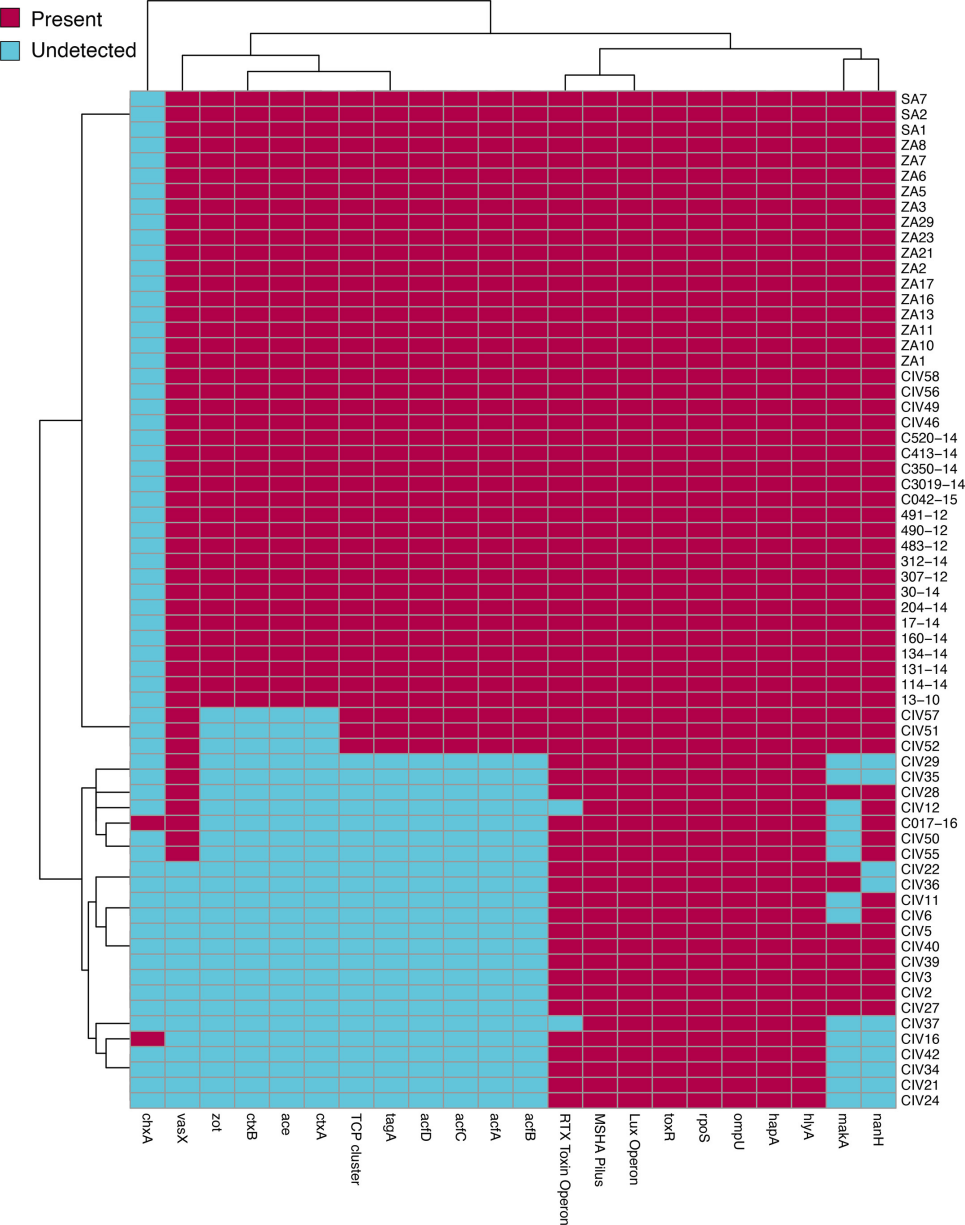
Binary heatmap showing the presence and absence of virulence traits among the study *V. cholerae* isolates. The heatmap displays the presence (raspberry) and absence (soft cyan) of specific virulence genes across the study isolates. Each row represents a sample, and each column represents a gene of interest, as identified via Pathogenwatch and TheiaProk (File S6). The virulence genes are as follows: *rpo*S, a stress gene involved in adhesion; *omp*U, an outer membrane protein also involved in adhesion; MSHA Pilus, a pili structure that promotes adhesion to host surfaces; *hap*A, a haemagglutinin/protease gene that facilitates bacterial invasion and immune evasion; *hly*A, a haemolysin gene that contributes to cytotoxicity; *tox*R, a transmembrane transcriptional activator that regulates the production of virulence factors, including cholera toxin; *ctx*A and *ctx*B, the cholera toxin genes responsible for cholera toxin production; ace, a toxin gene associated with cholera enterotoxin; *acf*A, *acf*B, *acf*C and *acf*D, colonisation factor genes necessary for intestinal colonisation; *zot*, a toxin gene that increases intestinal permeability; *chx*A, a toxin gene; and *vas*X, a gene encoding a toxin involved in the type VI secretion system, aiding bacterial virulence.

In line with their clinical origin, the *V. cholerae* O1 isolates were confirmed to carry the canonical virulence factors defining the toxigenic pathotype. These included the haemagglutinin gene *hap*A, known for its cytotoxic and mucinolytic activities [51], along with the toxin genes ace, *zot* and *hly*A (haemolysin) [52]; *tox*R, the transmembrane transcription factor that regulates the production of virulence factors in *V. cholerae* [53]; the cholera toxin genes *ctxA* and *ctxB*, which produce the ‘rice-water’ stools characteristic of cholera [54,55]; the colonisation operon *acfA-D*; and the toxin co-regulated pilus cluster [56] (Fig. 6). These virulence determinants were not detected in any of the non-O1 isolates. By contrast, the cholix toxin gene, *chxA* (an exotoxin) [57], was found in 8% (2 out of 24) of the environmental isolates but was absent from all clinical isolates. A previous study found *chxA* in both O1 (clinical) and non-O1 (environmental) strains [58]. The complete list of virulence traits detected among the study *Vibrio* isolates is provided in File S6.

## Discussion

This study demonstrates the utility of ONT sequencing for enhancing genomic surveillance of *V. cholerae* in Africa, a critical capability for networks like PulseNet Africa. By applying ONT sequencing, we were able to generate high-resolution data on the genetic diversity, AMR profiles and virulence traits of *V. cholerae* isolates from multiple African countries. This approach has significant potential for strengthening regional surveillance efforts for foodborne pathogens, including *V. cholerae*, as well as other critical pathogens under PulseNet Africa’s remit. The ability to rapidly generate and analyse whole-genome sequences using ONT in resource-limited settings could enhance the early detection of outbreaks, track the spread of AMR and improve public health responses across the continent.

Among the clinical isolates analysed, three non-O1 strains were retrieved from cases presenting with cholera-like symptoms. In addition, several study isolates were misidentified as *V. cholerae*, including non-*Vibrio* species such as *K. quasipneumoniae*, *E. hormaechei* and *A. enteropelogenes*. These findings underscore a significant diagnostic challenge: pathogens causing cholera-like disease are frequently misidentified as *V. cholerae* by conventional microbiological methods. Previous studies in Africa and elsewhere have suggested that certain enteric bacteria can cause cholera-like diarrhoeal infections, leading to their misattribution to *V. cholerae* during cholera outbreaks [59,62]. This issue is further underscored by findings from a large cholera outbreak in Malawi, where Chaguza *et al.* identified non-*V. cholerae* species in ~28% of cases (19 out of 68) [44].

The identification of clinically relevant *Vibrio* species, such as *V. fluvialis*, *V. furnissii* and *V. navarrensis*—all of which cause gastrointestinal illness [63]—is particularly noteworthy in the context of horizontal gene transfer among *Vibrio* species [64]. For instance, *V. mimicus*, though a non-*cholerae* species, can harbour the cholera toxin (*ctx*) gene [65], highlighting the potential for genetic exchange that may influence virulence. Similarly, *V. fluvialis* has been shown to possess an El Tor-like haemolysin (*hly*A), a gene associated with pathogenicity [66], underscoring the importance of monitoring these species for genetic traits relevant to outbreaks and public health.

From this study, the *V. furnissii* isolate was recovered from a clinical stool specimen, while *V. fluvialis* and *V. navarrensis* isolates were all sourced from sewage samples. These findings emphasise the necessity of accurate genomic characterisation and expanded surveillance for *Vibrio* species, especially given the challenges of speciation due to phenotypic heterogeneity even within a single *Vibrio* species. Genomic surveillance will be critical to improving pathogen detection, understanding their evolutionary dynamics and mitigating risks posed by emerging strains.

An earlier comprehensive survey of global *V. cholerae* genomic diversity by Weill *et al.* included strains from Côte d’Ivoire (*n*=29, spanning 1970–2006), Zambia (*n*=26, spanning 1996–2012) and Ghana (*n*=8, spanning 1970–2014) [11]. Both the Weill *et al*. study and subsequent genomic investigations have documented the circulation of multiple African lineages across the region, including AFR5, AFR10, AFR13 and AFR15 in Zambia [9,11, 15, 67], and AFR1, AFR9 and AFR12 in Ghana and Côte d'Ivoire [11,36]. By incorporating more recent isolates spanning 2010–2024, our analysis provides genomic insights on lineage circulation patterns.

Consistent with contemporary African cholera epidemiology, our phylogenetic analysis confirms that the majority of clinical isolates belong to the seventh Pandemic El Tor (7PET) lineage [4,8, 9, 11, 15, 46]. However, we also uncovered significant genetic diversity among the isolates, with several forming distinct clades within established pandemic lineages, which points to ongoing local transmission dynamics.

The overall genetic diversity was further underscored by two key findings. First, two ST75-2LV strains from our study clustered closely with the US Gulf Coast *V. cholera*e O1 clone [42], consistent with recent reports from South Africa. Second, the environmental isolates displayed considerable diversity, comprising five known and six potentially novel STs. Collectively, these findings demonstrate the importance of continuous, denser, high-resolution genomic surveillance for monitoring both the fine-scale evolution of endemic lineages and the presence of other diverse pathogen sources in the environment.

The clustering of non-O1 isolates into separate groups, particularly those recovered from sewage samples, indicates a divergence in environmental strains from those driving outbreaks. At least in our study, environmental strains do not appear to seed outbreaks, as they are genetically quite divergent from the clinical strains, although they harbour some key virulence traits. Further investigation into the role of environmental reservoirs as potential contributors to future outbreaks and their broader role in cholera epidemiology is warranted [68,71].

The AMR gene profile of the study isolates is consistent with the established resistance patterns of the seventh Pandemic El Tor (7PET) lineage circulating since the 2000s. The near-universal presence of *dfr*A alleles and the double mutations in *gyr*A (S83I) and *par*C (S85L) are characteristic of this lineage, conferring resistance to trimethoprim-sulphamethoxazole and quinolones, respectively [4,11, 72]. Consequently, all O1 isolates were genotypically multi-drug resistant (MDR), exhibiting resistance to at least three antibiotic classes, which aligns with contemporary reports from other African regions [43,47].

While these findings confirm that quinolones and other frontline antibiotics are likely ineffective for cholera treatment in the region, resistance determinants for azithromycin and rifampicin were notably absent. The preservation of azithromycin susceptibility is crucial and supports its continued use as a first-line therapeutic option [4,6]. The primary emerging threat now appears to be the acquisition of MDR IncC plasmids, which are responsible for high-level drug resistance in 7PET AFR13 isolates recently reported from Eastern Africa [4,11, 72]. The absence of these plasmids in our isolates is a key finding, but the regional threat they pose underscores the necessity of continued genomic surveillance to monitor their potential spread.

## Limitations

Our study is subject to several important limitations that may affect the interpretation of our findings. Historical and contemporary undersampling in the studied countries represents a significant constraint on our phylogenetic analyses and epidemiological interpretations. As demonstrated in previous genomic studies, insufficient or geographically biassed sampling can lead to an incomplete or inaccurate picture of transmission dynamics [9]. This limitation is particularly relevant given the historically limited genomic surveillance capacity in sub-Saharan Africa, which may have led to systematic underrepresentation of certain lineages or transmission networks, and underscores the need for enhanced surveillance and more systematic sampling strategies to provide a complete picture of *V. cholerae* evolution and transmission in the region.

A major limitation of this study is that the sample collection relied on available archived and viable isolates from four countries. As a result, the collection may not fully represent the diversity of circulating *V. cholerae* strains in the region during the study period. The lack of phenotypic antimicrobial susceptibility test data is another limitation. Although we were able to analyse the genotypic profiles of AMR, phenotypic data would have provided direct evidence of resistance patterns in the isolates. Unfortunately, logistic constraints did not allow us to conduct phenotypic testing, which limits the study’s ability to fully assess the clinical implications of the detected resistance genes.

## Conclusion

Our study demonstrates the value of using WGS to uncover the genomic diversity, AMR profiles and virulence traits of *V. cholerae* strains in Africa. Further work is needed to develop standardised QC metrics and standard operating procedures for handling ONT sequencing data within PulseNet Africa and its sister networks. Establishing these protocols will ensure data consistency, reliability and comparability across labs, thereby strengthening the regional genomic surveillance framework for *V. cholerae* and other pathogens.

The identification of clonal expansion within certain lineages, characterised by high levels of AMR genotypes in clinical isolates, provides essential information for improving cholera surveillance and response strategies. Given the high prevalence of resistance to critical antibiotics such as trimethoprim and quinolones and the potential for further AMR evolution, it is imperative to reassess treatment guidelines and enhance genomic monitoring.

## Supplementary material

10.1099/mgen.0.001586 Supplementary Material 1.

10.1099/mgen.0.001586 Supplementary Material 2.

## Abbreviations

AMR: antimicrobial resistance
CDC: Centers for Disease Control and Prevention
CLIMB: Cloud Infrastructure for Microbial Bioinformatics
MDR: multi-drug resistant
MLVA: multiple-locus variable number tandem repeat analysis
MRCG at LSHTM: Medical Research Council Unit The Gambia at London School of Hygiene and Tropical Medicine
NICD: National Institute for Communicable Diseases
ONT: Oxford Nanopore Technology
WGS: whole-genome sequencing
WHO: World Health Organization
