# Supplementary material for: Genomic diversity and antimicrobial resistance of Vibrio cholerae isolates from Africa: a PulseNet Africa initiative using nanopore sequencing to enhance genomic surveillance
Source: Microb Genom. 2025 Dec 10;11(12):001586. doi: 10.1099/mgen.0.001586 (PMC12692737; doi:10.1099/mgen.0.001586)
Supplement: Supplementary Material 1. [file mgen-11-01586-s001.pdf]

Genomic Diversity and Antimicrobial Resistance of *Vibrio cholerae* Isolates from Africa: A PulseNet Africa Initiative Using Nanopore Sequencing to Enhance Genomic Surveillance

Ebenezer Foster-Nyarko, Shola Able-Thomas, Nana Eghele Adade, Rexford Adade, Jean Claude Blessa Anne, Loretta Antwi, Yaya Bah, Gifty Boateng, Heather Carleton, David Chaima, Roma Chilengi, Kalpy Julien Coulibaly, Firehiwot Abera Derra, Dwayne Didon, Cheelo Dimuna, Mireille Dosso, Momodou M. Drammeh, Sana Ferjani, Kathryn E. Holt, Rohey Jatta, John Bosco Kalule, Abdoulie Kanteh, Hortense Faye Kette, Dam Khan, N'da Kouame Nazaire Kouadio, Christine Lee, Hamakwa Mantina, Gillan Mulenga, John Mwaba, Fatou Nyang, Godfred Owusu-Okyere, Jessica Rowland, Aissatou Seck, Abdul Karim Sesay, Anthony Smith, Peyton Smith, Djifahamaï Soma, Nomsa Tau, Pierrette Landrie Simo Tchuinte, Peggy-Estelle Maguiagueu Tientcheu, Chalwe Sokoni, Sabine N'dri Vakou, Delfino Vubil; PulseNet Africa

File S1

1. Methods Used for Isolation and Characterisation of *Vibrio* Isolates

Isolation Method:

For stool samples: Stool samples from suspected cholera patients were collected in clean containers and processed following standard microbiological methods. Samples were plated on Thiosulfate Citrate Bile Salts Sucrose (TCBS) agar (Oxoid, UK), both directly and after 6 hours of incubation in alkaline peptone water (APW). Plates were incubated at 37°C for 18–24 hours. Single yellow colonies indicative of *V. cholerae* were selected, subcultured on Mueller-Hinton agar (Oxoid, UK), and incubated at 37°C for 24 hours.

For wastewater samples: 1 ml of enriched wastewater in 9 ml of Eau Peptonnée Alcaline (EPA) was incubated at 37°C for 24 hours. Samples were plated on TCBS agar, nutritive agar, and enriched in alkaline peptone and Mueller Kauffman broths. Yellow, "Chinese hat"-shaped colonies on TCBS agar were subcultured on Nutritive Agar (GNA) and incubated at 37°C for 24 hours, followed by Gram staining and biochemical tests (oxidase, motility, and Leminor's test).

Characterization Techniques:

An oxidase test was performed on colonies grown on Mueller-Hinton agar. Oxidase-positive colonies were serotyped using O1 polyvalent, O1 Inaba, O1 Ogawa, and O139 antisera (Mast Diagnostics, UK). Colonies agglutinating with O1 polyvalent and specific antisera were designated as *V. cholerae* O1 Inaba or Ogawa. Biochemical tests confirmed characteristics typical of *Vibrio* spp., such as Gram-negative curved bacilli, oxidase positivity, and other traits: citrate +, urea -, indole -, gas +, H_2S -, LDC +, and LDA -.

Serogrouping and serotyping were confirmed using Vibrio cholerae O1 antiserum (Bio-Rad, France).

2. Epidemiological Data on Outbreaks

2023/2024 Cholera Outbreak in Zambia:

- **Total Cases:** 23,356 cases with 740 deaths
- **Outbreak Description:** The index case for the 2023/2024 cholera outbreak was reported in Lusaka Province in October 2023, a cholera-prone region. From October 2023 to June 2024, cases were reported in 72 of 116 districts across all 10 provinces of Zambia. In January 2024, an Oral Cholera Vaccine (OCV) campaign targeted high-burden areas, achieving 99% coverage (1,870,375/1,888,112 of the target population) [2, 3].

2014–2015 Cholera Outbreak in Côte d'Ivoire:

The epidemic began on October 8, 2014, on an island south of Abidjan and spread inland, reaching towns over 200 km away. The last cases were reported in February 2015 in interior health districts. A total of 456 suspected cases were recorded, with 109 confirmed as *Vibrio cholerae* O1 El Tor Ogawa.

- **Confirmed Cases by Month:**

Month	Year	Number of *Vibrio* isolates
October	2014	15
November	2014	37
December	2014	27
January	2015	28
February	2015	3

References

1. Centers for Disease Control and Prevention. Laboratory methods for the diagnosis of epidemic dysentery and cholera. In: Laboratory Methods for the Diagnosis of Epidemic Dysentery and Cholera. CDC: Atlanta, GA, 1999.
2. Zambia National Public Health Institute. https://w2.znphi.co.zm/resources/
3. UNICEF Zambia. Flash Update: Cholera, March 7, 2024. https://www.unicef.org/media/153511/file/Zambia-Flash-Update-Cholera-07-March-2024.pdf

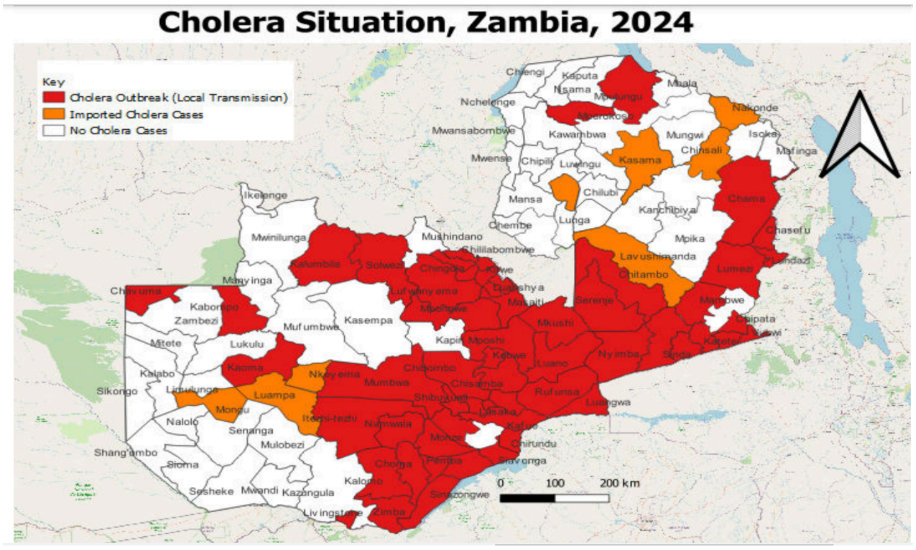

References

1. Centers for Disease Control and Prevention. Laboratory methods for the diagnosis of epidemic dysentery and cho lera. In: CDC, editor. Laboratory Methods for the Diagnosis of Epidemic Dysentery and Cholera. CDC: Atlanta, GE, 1999
2. https://w2.znphi.co.zm/resources/
3. https://www.unicef.org/media/153511/file/Zambia-Flash-Update-Cholera-07-March-2024.pdf

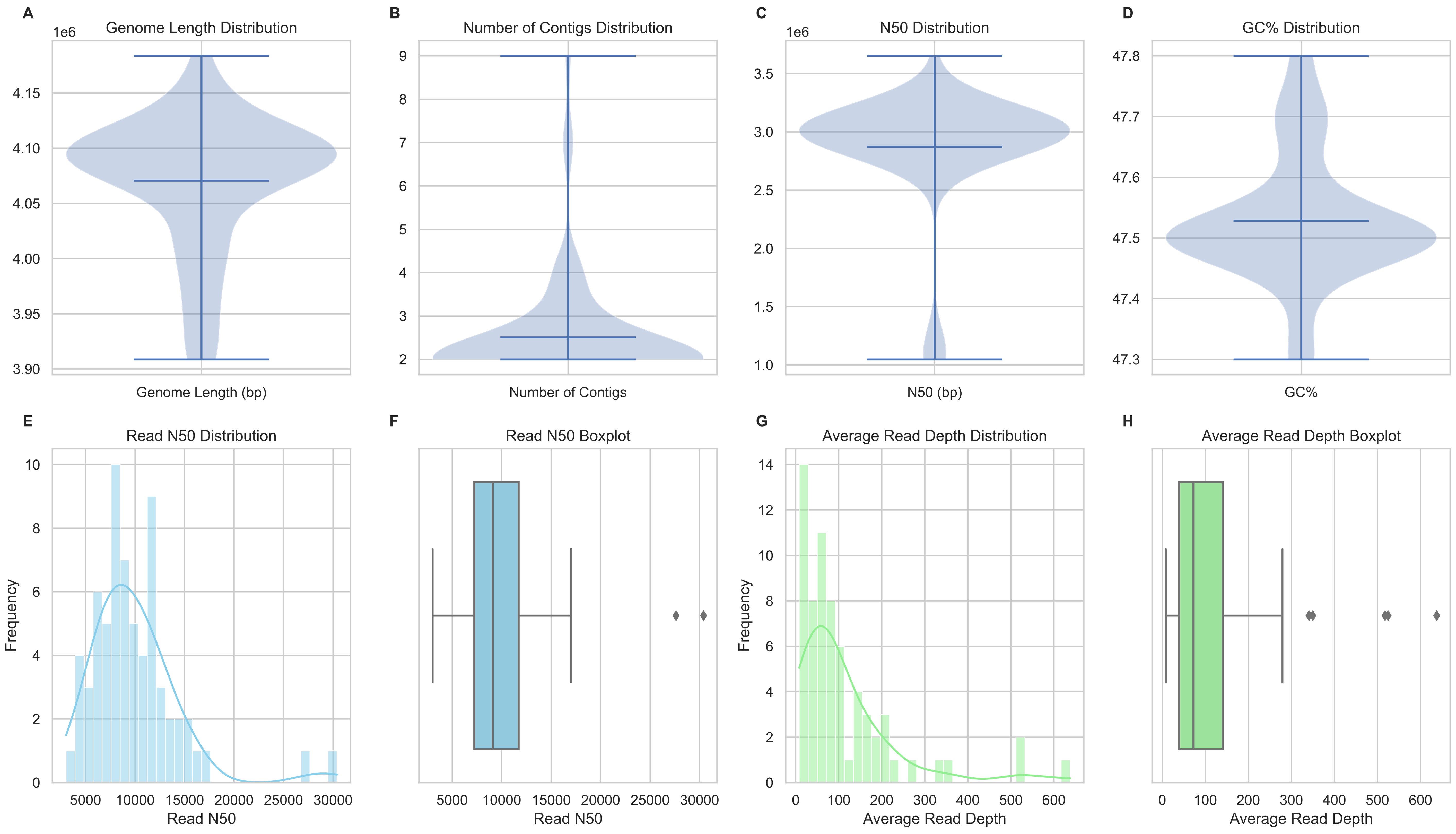

Figure S2. A Neighbour-Joining tree illustrating the phylogenetic placement of the study isolates ST-69-4LV and ST-75-2LV. The tree was reconstructed using Vibriowatch (Reference #24) and incorporates the collection of isolates published by Chun et al. (Reference #31). Study isolates are represented by circular tips with red labels, while reference isolates from the Chun collection are represented by square tips and black labels.
The soft brick red dashed rectangle highlights isolates belonging to the current global "7th pandemic" (7PET) clade, whereas the teal rectangle marks the broader Phylocore Group 1 (PG-1) clade. Notably, the two ST-75-2LV study isolates cluster closely with NCTC_8457, MAK757, and BX330286, which are sometimes referred to as 'pre-7PET' or 'Gulf-Coast-like' isolates.

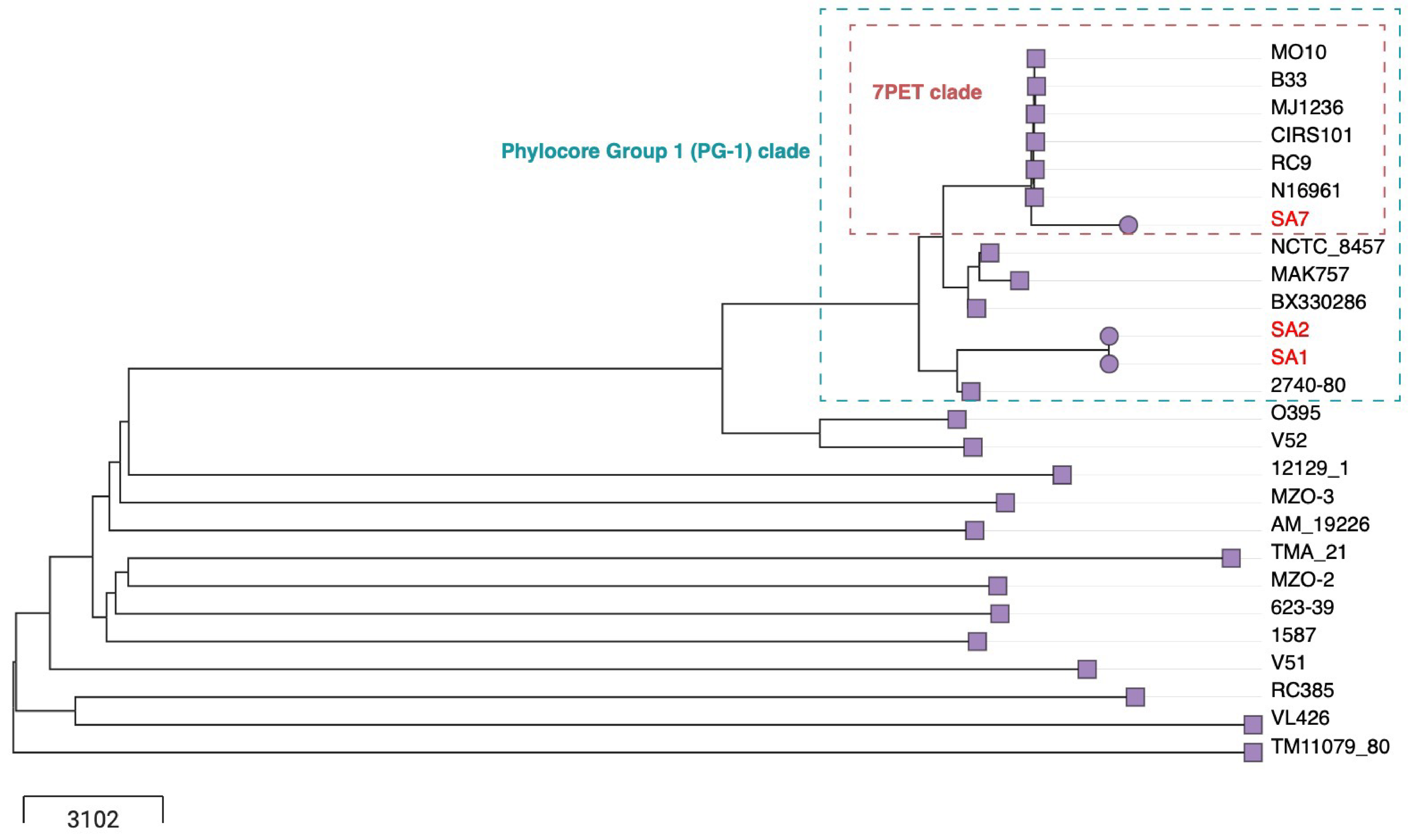

Figure S2. A Neighbour-Joining tree illustrating the phylogenetic placement of the study isolates ST-69-4LV and ST-75-2LV. The tree was reconstructed using Vibriowatch (Reference #24) and incorporates the collection of isolates published by Chun et al. (Reference #31). Study isolates are represented by circular tips with red labels, while reference isolates from the Chun collection are represented by square tips and black labels.
The soft brick red dashed rectangle highlights isolates belonging to the current global "7th pandemic" (7PET) clade, whereas the teal rectangle marks the broader Phylocore Group 1 (PG-1) clade. Notably, the two ST-75-2LV study isolates cluster closely with NCTC_8457, MAK757, and BX330286, which are sometimes referred to as 'pre-7PET' or 'Gulf-Coast-like' isolates.

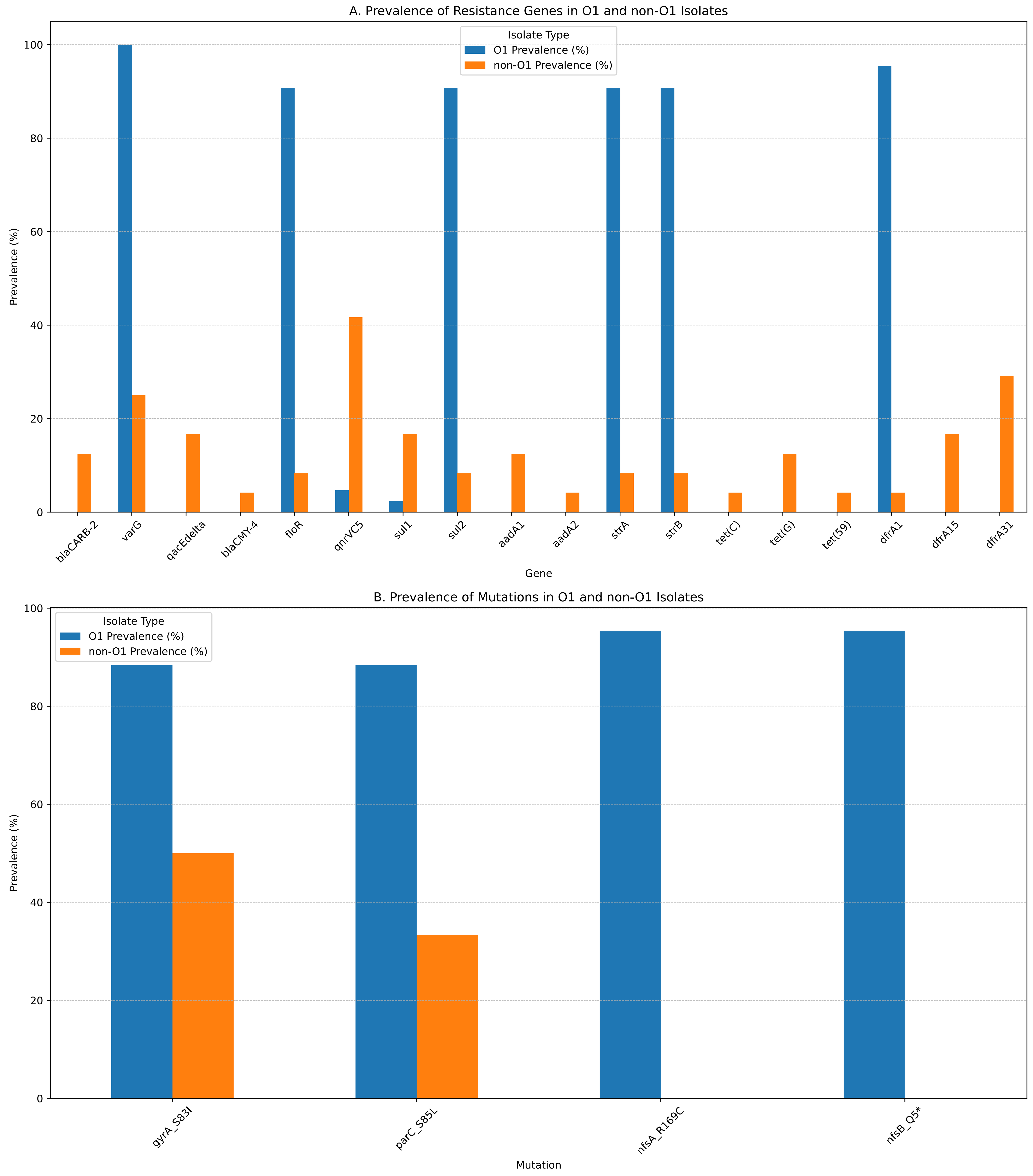

Figure S3. Prevalence of resistance genes and mutations in O1 vs non-O1 isolates. (**A**) The bar plot shows the prevalence of resistance genes across O1 and non-O1 isolates. The blue bars represent the prevalence of resistance genes in O1 isolates, while the orange bars represent the prevalence in non-O1 isolates. The genes are grouped by antibiotic resistance classes, including penicillins (blaCARB-2), carbapenems (varG), third-generation cephalosporins (blaCMY-4), chloramphenicol (floR), aminoglycosides (aadA1, aadA2, strA, strB), tetracyclines (tet(C), tet(G), tet(59), and trimethoprim (dfrA1, dfrA15, dfrA31).
(**B**) The bar plot shows the prevalence of key mutations associated with antibiotic resistance in O1 and non-O1 isolates. The blue bars represent the prevalence in O1 isolates, and the orange bars represent non-O1 isolates. The mutations include those associated with quinolone resistance (gyrA_S83I, parC_S85L) and furazolidone resistance (nfsA_R169C, nfsB_Q5*). Data labels indicate the precise prevalence percentages.
